# Localization and subcellular association of Grapevine Pinot Gris Virus in grapevine leaf tissues

**DOI:** 10.1007/s00709-017-1198-5

**Published:** 2017-12-22

**Authors:** Giulia Tarquini, Paolo Ermacora, Gian Luca Bianchi, Francesca De Amicis, Laura Pagliari, Marta Martini, Alberto Loschi, Pasquale Saldarelli, Nazia Loi, Rita Musetti

**Affiliations:** 1 Department of Agricultural, Food, Environmental and Animal Sciences, University of Udine, via delle Scienze, 206, 33100 Udine, Italy; 2 ERSA, Servizio fitosanitario e chimico, ricerca, sperimentazione ed assistenza tecnica, via Sabbatini, 5, Pozzuolo del Friuli, 33050 Udine, Italy; 3 CNR-Institute for Sustainable Plant Protection, via Amendola, 165/A, 70126 Bari, Italy

**Keywords:** *Betaflexiviridae*, GPGV, Grapevine, Transmission electron microscopy, Virus

## Abstract

Despite the increasing impact of Grapevine Pinot gris disease (GPG-disease) worldwide, etiology about this disorder is still uncertain. The presence of the putative causal agent, the *Grapevine Pinot Gris Virus* (GPGV), has been reported in symptomatic grapevines (presenting stunting, chlorotic mottling, and leaf deformation) as well as in symptom-free plants. Moreover, information on virus localization in grapevine tissues and virus-plant interactions at the cytological level is missing at all. Ultrastructural and cytochemical investigations were undertaken to detect virus particles and the associated cytopathic effects in field-grown grapevine showing different symptom severity. Asymptomatic greenhouse-grown grapevines, which tested negative for GPGV by real time RT-PCR, were sampled as controls. Multiplex real-time RT-PCR and ELISA tests excluded the presence of viruses included in the Italian certification program both in field-grown and greenhouse-grown grapevines. Conversely, evidence was found for ubiquitous presence of *Grapevine Rupestris Stem Pitting-associated Virus* (GRSPaV), *Hop Stunt Viroid* (HSVd), and *Grapevine Yellow Speckle Viroid 1* (GYSVd-1) in both plant groups. Moreover, in every field-grown grapevine, GPGV was detected by real-time RT-PCR. Ultrastructural observations and immunogold labelling assays showed filamentous flexuous viruses in the bundle sheath cells, often located inside membrane-bound organelles. No cytological differences were observed among field-grown grapevine samples showing different symptom severity. GPGV localization and associated ultrastructural modifications are reported and discussed, in the perspective of assisting management and control of the disease.

**Electronic supplementary material:**

The online version of this article (10.1007/s00709-017-1198-5) contains supplementary material, which is available to authorized users.

## Introduction

Grapevine Pinot gris disease (GPG-disease) first occurred in 2003 in Northern Italy, when symptoms reminiscent of viral diseases were initially detected on cv. Pinot gris (Giampetruzzi et al. 2012), and then on cvs. Traminer and Pinot noir (Giampetruzzi et al. 2012). The visible alterations in the field appeared on leaves soon after sprouting and included stunting, chlorotic mottling mosaic, and leaf deformation, and later, a decrease in yields. Very frequently, after passing through a period of bearing vegetation with symptoms, diseased plants recovered from the syndrome and their new vegetation developed normally, masking the symptomatic tissue, and making visual symptom detection difficult during summer (Bianchi et al. 2015).

Next-generation sequencing approaches and small-RNA analyses have been applied to symptomatic grapevine tissues and led to the identification of a new virus, provisionally named *Grapevine Pinot Gris Virus* (GPGV; Giampetruzzi et al. 2012).

GPGV has a positive-sense single-stranded RNA genome. It has been included in the order *Tymovirales* and in the recently established *Betaflexiviridae* virus family, genus *Trichovirus* (Martelli 2014), due to a significant genome structure similarity to members of this taxon, such as *Grapevine Berry Inner Necrosis Virus* (GINV) (Giampetruzzi et al. 2012).

GPGV occurs in different grapevine cultivars, such as Pinot gris, Pinot noir, Traminer, Tocai, and Glera, and it can be present both in symptomatic and asymptomatic plants (Bianchi et al. 2015; Saldarelli et al. 2015). In fact, the virus was detected by real-time RT-PCR also in asymptomatic plants, further complicating the still debated disease etiology. The wide difference in symptom severity and the molecular detection of the virus in asymptomatic plants indicate the lack of an unambiguous correlation between the occurrence of the syndrome and the newly described virus.

GPGV is widely distributed in Italy (Giampetruzzi et al. 2012; Raiola et al. 2013; Bertazzon et al. 2016a; Bianchi et al. 2015; Gentili et al. 2017) and in many other European countries such as Slovakia and the Czech Republic (Glasa et al. 2014), Poland (Eichmeier et al. 2017), Slovenia (Pleško et al. 2014), France (Beuve et al. 2015), and Greece (Martelli 2014). GPGV has also been reported in South Korea *(*Jung et al. 2013), Turkey (Gazel et al. 2015), China (Fan et al. 2015), the USA (Al Rwahnih et al. 2015), and Canada (Poojari et al. 2016).

Despite the increase in the number of reports describing GPG-disease in vineyards worldwide, the literature lacks information on virus localization in grapevine tissues and virus-plant interactions at the cytological level. Given that virus localization in the host plants is related to insect vector feeding features and ecology (Whitfield et al. 2015), a description of the relationship between GPGV and grapevine tissues at the ultrastructural level may have important implications for further studies related to disease transmission and epidemiology.

The aim of this work is to provide an accurate description of the localization of virus inside grapevine tissues and to evaluate the cytopathic modifications in symptomatic and asymptomatic plants. The observations provide first insights into the interactions of GPGV with grapevine tissues.

## Materials and methods

### Plant material and symptom evaluation

A vineyard of cv. Pinot gris, clone VCR5 grafted on Kober 5BB, established in 2003 and located in Farra d’Isonzo (Friuli Venezia Giulia, north-eastern Italy), was monitored for the presence of viral-like symptoms for four consecutive vegetative seasons since 2013.

**Fig. 1:**
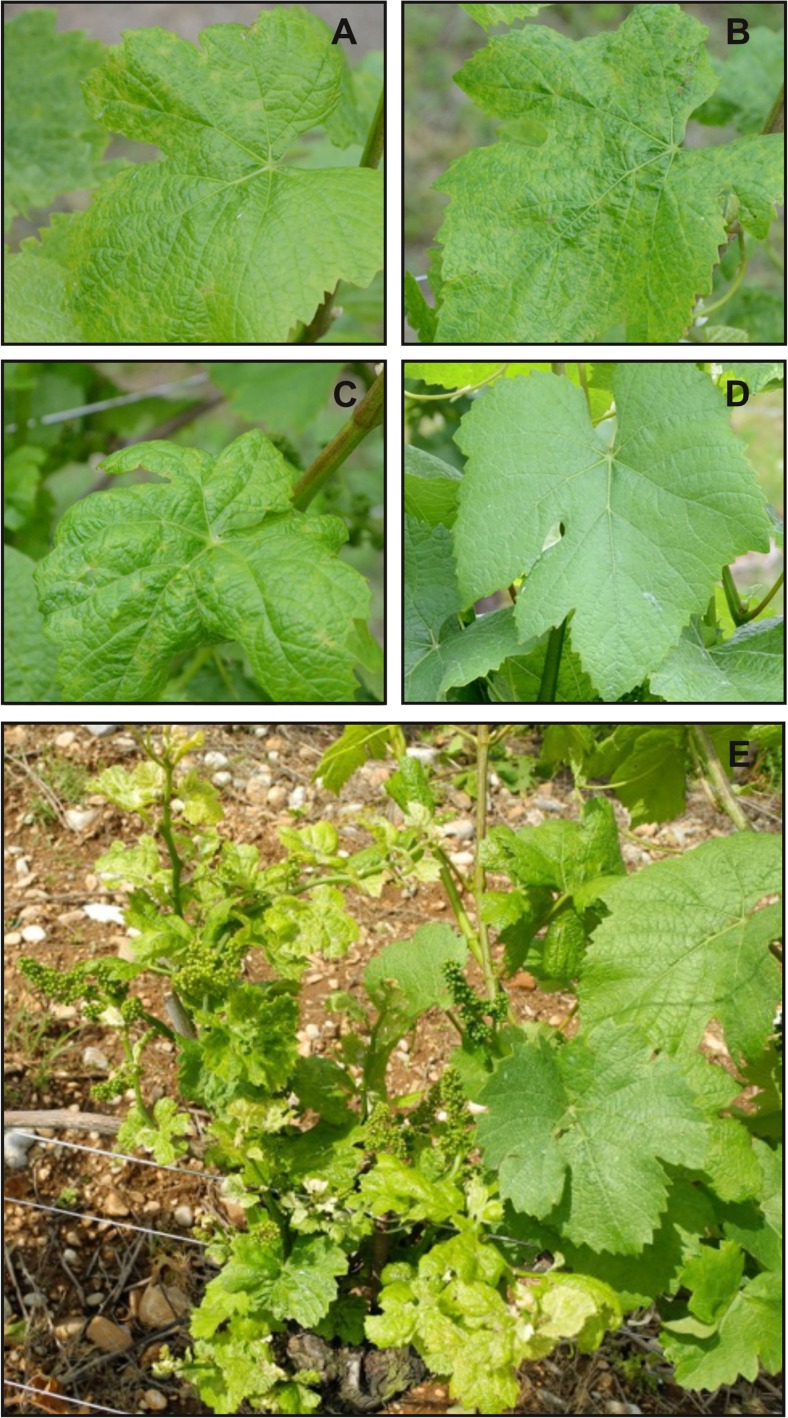
Pinot gris grapevines showing symptoms with different severity. **a** Leaf from a grapevine showing mild symptoms, i.e., chlorotic mottling, without puckering, and malformations. **b** A leaf from moderately symptomatic grapevine showing wide chlorotic mottling, mild deformation, and puckering. **c** Wide chlorotic leaf mottling with severe leaf deformation and puckering are visible on a leaf from a severely symptomatic grapevine. **d** A leaf from an asymptomatic grapevine is perfectly formed. **e** Grapevine with severe symptoms (on the left) close to a symptomless one (on the right). Besides chlorotic leaf mottling, severe leaf deformation, and puckering, a grapevine with severe leaf symptoms shows significant reduction in growth and development in comparison to an asymptomatic plant

A total of 11,000 grapevines at the BBCH 53–55 phenological stages were surveyed for symptom expression every year. Among them, 30 randomly selected plants were tested three times per year, since 2013, by real-time RT-PCR to assess the GPGV presence. Plants were grouped into four classes according to symptom severity in the field: mild, moderate, severe (Fig. 1a–e), and symptomless (Fig. 1d). As reported in Fig. 1a, individuals with limited presence of chlorotic mottling on leaves without puckering and malformations were defined as mildly symptomatic plants. Moderately symptomatic grapevines (Fig. 1b) showed widespread chlorotic mottling and mild leaf deformation and puckering. Finally, plants with widespread chlorotic leaf mottling with severe leaf deformation and puckering were classified as severely symptomatic plants (Fig. 1c, e).

For each class, five grapevines, which showed the same respective symptoms in 2014 and 2015, were collected at the BBCH 53–55 phenological stages and processed as required by the different protocols described in this paper. Dormant canes and leaves were tested by real-time RT-PCR and ELISA for the viruses included in the Italian certification program (Bertazzon et al. 2002), namely *Grapevine Viruses* A and B (GVA, GVB), *Grapevine Fleck Virus* (GFkV), *Grapevine Leafroll-associated Viruses* 1, 2, 3 (GLRaV-1, GLRaV-2, GLRaV-3), *Grapevine Fanleaf Virus* (GFLV), and *Arabis Mosaic Virus* (ArMV).

Leaves collected from the same grapevines were further analyzed by multiplex real-time RT-PCR for the detection of viruses and viroids reported in Pinot gris tissues simultaneously with GPGV (Giampetruzzi et al. 2012): *Grapevine Rupestris Stem Pitting-associated Virus* (GRSPaV), *Grapevine Rupestris Vein Feathering Virus* (GRVFV), *Grapevine Syrah Virus 1* (GSyV-1), *Hop Stunt Viroid* (HSVd) and *Grapevine Yellow Speckle Viroid* 1 (GYSVd-1). Moreover, GPGV molecular detection and transmission electron microscopy (TEM) analyses were performed on the same material. Canes and leaves from five asymptomatic Pinot gris grapevines, grown and maintained in a greenhouse, which were negative to GPGV by real-time RT-PCR (named greenhouse-grown grapevines below), were also sampled and used as controls.

### Detection of GPGV in grapevine tissues

#### RNA extraction

Total RNA was extracted from leaf veins and woody canes of grapevine sampled in the field and in the greenhouse. Leaf veins (0.5 g) were collected and ground into fine powder in the presence of liquid nitrogen; for dormant canes, 1 g of subcortical vascular tissue was scraped using a semi-automated homogenizer (Turner-Lavorazioni meccaniche Linzi Mauro, Udine, Italy) and then transferred to a plastic bag with a filter (Bioreba, Reinach, Switzerland).

All samples were homogenized with 5 ml of lysis buffer containing 4 M guanidine isothiocyanate, 0.2 M sodium acetate, pH 5.0, 25 mM EDTA, 2.5% (wt/vol) PVP-40, and 1% (vol/vol) sodium metabisulphite, added just before use (MacKenzie et al. 1997). An aliquot of 1.5 ml of homogenate was transferred to a 2-ml microcentrifuge tube and centrifuged for 6 min at 12,000 rpm. One milliliter of supernatant was collected in a 2-ml Eppendorf tube, mixed with 100 μl of 20% (wt/vol) sarkosyl and incubated for 10 min at 70 °C in a water bath. Samples were then transferred into a QIAshredder filtration column and RNA was purified with an RNeasy plant mini kit (Qiagen, Hilden, Germany) according to the manufacturer’s recommendations. The final elution volume was set to 50 or 100 μl for RNA extracted from leaves or woody canes, respectively, and eluted RNA was stored at − 80 °C until further use.

#### Molecular assay for GPGV detection in grapevine tissues

GPGV detection was performed by two-step real-time RT-PCR. Samples were assayed for the presence of the coat protein gene using the specific primers GPgV504-F (5′-GAATCGCTTGCTTTTTCATG-3′) and GPgV588-R (5′-CTACATACTAAATGCACTCTCC-3′), according to Bianchi et al. (2015).

#### cDNA synthesis

First-strand cDNA synthesis was performed using recombinant *Moloney murine leukemia virus* (MMLV) reverse transcriptase (Promega Corporation, Madison, WI, USA) and a blend of random hexamer primers (Roche Diagnostic, Indianapolis, IN, USA). The first phase of the reaction was carried out by incubating 5 μl of total RNA with 0.5 ng/μl of random hexamer primers for 5 min at 70 °C. Samples were kept on ice for 10 min. The final volume of the second phase was 25 μl per reaction including 5 μl of M-MLV 5X reaction buffer, 2.5 mM dNTPs, 25 units of recombinant RNasin ribonuclease inhibitor (Promega Corporation, Madison, WI, USA), and 200 units of M-MLV reverse transcriptase enzyme (Promega Corporation, Madison, WI, USA). Samples were incubated for 1 h at 37 °C and the resulting cDNA was stored at − 20 °C.

#### Real-time PCR

Real-time PCR was performed in 15 μl reaction volume mixtures with 1 μl of cDNA, 7.5 μl of SsoFast EvaGreen Supermix (Bio-Rad, Hercules, CA, USA) and 2.5 mM of each primer (GPgV504-F and GPgV588-R). The following thermal protocol was used: 98 °C for 2 min; 45 cycles of denaturation at 98 °C for 5 s and annealing/extension at 60 °C for 5 s; final denaturation at 95 °C for 1 min and final extension at 65 °C for 1 min. Every plate included a non-template and a positive (cDNA from GPGV-infected plant) control. For each sample, three technical replicates were performed.

All reactions were performed on a CFX96 real-time system (Bio-Rad, Hercules, CA, USA) and amplification data were analyzed using the CFX Manager Software 2.0 (Bio-Rad). To allow comparability between assays, the baseline threshold was always set to 100 RFU (relative fluorescence units) and samples were considered positive for GPGV when threshold cycle (Ct) values were < 35, with values among 30 and 34 considered as low positive (Vončina et al. 2017). To compare different Ct values among samples with different symptom severity, statistical analyses were performed with the InStat GraphPad software package (La Jolla, CA, USA) using one-way ANOVA and Tukey-Kramer multiple comparisons test as post hoc test. A *P* value < 0.005 was considered statistically significant.

### Detection of viruses included in the Italian certification program

#### Multiplex real-time RT-PCR

To evaluate the sanitary status of the 25 grapevines, further assays were performed by real-time RT-PCR according to the methods developed by Bianchi et al. (2010). One-step multiplex real-time RT-PCR was used for the detection of GVA, GFLV, ArMV, GLRaV-1, and GLRaV-3. Five microliters of RNA was added to 12.5 μl of 2X QuantiFast multiplex RT-PCR Master mix without ROX (Qiagen, Hilden, Germany) supplemented with 0.25 μl of QuantiFast RT Mix (Qiagen, Hilden, Germany), 0.4 μM final concentration of each primer and 0.2 μM of the probes, and RNase-free water to a final volume of 25 μl. Multiplex one-step real-time RT-PCR was performed on a CFX96 real-time system (Bio-Rad, Hercules, CA, USA) using the following amplification conditions: 50 °C for 30 min, 95 °C for 5 min followed by 45 cycles of 95 °C for 5 s, and 60 °C for 30 s. All samples were analyzed at least twice and each run included a no template control, a negative control, and a positive control for each virus.

All real-time PCR data were analyzed using the CFX Manager software 2.0 (Bio-Rad, Hercules, CA, USA). Samples were considered positive for a mean Ct value < 30, with a baseline threshold set to 100 RFU in all PCR reactions (Bianchi et al. 2010).

#### ELISA

To complete and confirm the results obtained by multiplex one-step real-time RT-PCR, dormant canes and leaf samples were tested by indirect DAS-ELISA using commercial kits against different grapevine viruses (Agritest srl, Valenzano, Italy).

### Detection of other grapevine viruses and viroids

When GPGV was discovered by Giampetruzzi et al. (2012), three more viruses and two viroids were also detected in Pinot gris tissues: GRSPaV, GRVFV, GSyV-1 HSVd, and GYSVd-1. To disclose any interaction among these pathogens and GPGV in ultrastructural alterations of grapevine tissues, the 25 samples included in this study were further analyzed. Two duplex one-step real-time RT-PCR were performed for the simultaneous detection of GRVFV + GSyV-1 and HSVd + GYSVd-1, respectively, while a simplex one-step real-time RT-PCR was conducted for the detection of GRSPaV. Both duplex and simplex real-time RT-PCR were performed according to the protocol developed by Bianchi et al. (2010) previously described, using primers/probe combinations as described in Bianchi et al. (2015).

### Conventional transmission electron microscopy

From each plant, five leaves, coeval and similar in shape, were collected for ultrastructural analysis. Segments (3–4 mm in length) of leaf tissues including both vein tissue and surrounding parenchyma cells were fixed in 3% glutaraldehyde, rinsed in phosphate buffer (PB) 0.15 M, postfixed in 1% osmium tetroxide in 0.15 M PB for 2 h at 4 °C, dehydrated in ethanol and embedded in Epon-Araldite epoxy resin (Electron Microscopy Sciences, Fort Washington, PA, USA) according to the method described by Musetti et al. (2005). Ultrathin sections (60–70 nm) of about 60 resin-embedded samples from each field- or greenhouse-grown control plant were cut using an ultramicrotome (Reichert Leica Ultracut E ultramicrotome, Leica Microsystems, Wetzlar, Germany) and collected on 200 mesh uncoated copper grids. Sections were then stained with 3% uranyl acetate and 0.1% lead citrate (Reynolds 1963) and observed under a PHILIPS CM 10 (FEI, Eindhoven, The Netherlands) TEM, operated at 80 kV. Five non-serial cross-sections from each sample were analyzed.

### Immuno-cytochemical identification of GPGV in grapevine tissues

An immunogold labelling experiment was carried out to provide evidence that the virus detected with TEM observations was GPGV. Five leaves, coeval and similar in shape, were collected from two symptomatic and two asymptomatic grapevines, grown in the field, in which GPGV has been previously detected by real-time RT-PCR approach. Leaves from five greenhouse-grown grapevines were also collected and used as GPGV-negative controls.

The experiment was performed according to the protocol reported by Musetti et al. (2002), with minor modifications: samples were cut into small portions (6–7 mm in length), fixed 1 h in 0.2% glutaraldehyde, rinsed in 0.05 M PB pH 7.4, and dehydrated in graded ethanol series (25, 50, 75%, 30 min for each step) at 4 °C. After 1 h of the final 100% ethanol step, the samples were infiltrated in a hard-grade London Resin White (LRW, Electron Microscopy Sciences, Fort Washington, PA, USA) 100% ethanol mixture in the proportion 1:2 for 30 min, followed by LRW/ethanol 2:1 for 30 min, and 100% LRW overnight at room temperature (with a change 1 h after the start of the infiltration). The samples were embedded in beem capsules (Electron Microscopy Sciences, Fort Washington, PA, USA) using fresh LRW containing benzoyl peroxide 2% (*w*/*w*) according to manufacturer’s protocol and polymerized for 24 h at 50 °C.

Several ultrathin sections (60–70 nm) of about 40 LR-White-embedded samples from asymptomatic or symptomatic grapevines were cut using an ultramicrotome (Reichert Leica Ultracut E ultramicrotome, Leica Microsystems, Wetzlar, Germany) and collected on carbon/formvar-coated 400 mesh nickel grids (Electron Microscopy Sciences, Fort Washington, PA, USA). Unspecific binding sites were blocked placing grids carrying the sections on droplets of blocking solution, containing 0.05 M Tris-buffered saline (TBS), pH 7.6, and 1:30 normal goat serum (NGS) for 30 min. Grids were then incubated overnight with primary rabbit polyclonal antibody (Pab) against GPGV coat protein (CP), produced and characterized by Gualandri et al. (2015). The Pab was diluted in 0.05 M TBS, pH 7.6 containing 1:30 NGS. Control grids were incubated only in TBS/NGS solution without primary antibody. All grids were washed five times in 0.05 M TBS (for 3 min each one), treated for 1 h with secondary goat anti-rabbit antibody conjugated with colloidal 10 nm gold particles (GAR 10) (EM GAR G10 BBI solutions, Cardiff, UK) diluted in TBS, and then washed again as described above. Different dilutions of primary CP-Pab and GAR 10 were evaluated in order to obtain the best combination between each other, both on greenhouse and field-grown grapevine samples.

Sections were fixed in 2% glutaraldehyde for 5 min, then on 1% OsO_4_ for 15 min. After staining with 3% uranyl acetate and 0.1% lead citrate (Reynolds 1963), samples were observed under TEM, as reported above. Five non-serial cross-sections from each sample were analyzed.

## Results

### Plant material and symptom evaluation in field-grown grapevines

According to the symptoms present in the field-grown grapevines, disease prevalence (i.e., percentage of symptomatic plants in a given year, McRoberts et al. 2003) decreased from 64.0% in 2013 to 13.2% in 2016. Annual incidence of the disease (i.e., newly symptomatic plants per year, McRoberts et al., 2003) was 9.2% in 2014, and decreased to very low values in 2015 and 2016 (Table 1). In 2013, the presence of GPGV in the randomly sampled grapevines was 90%. The percentage reached up the 100% in 2014, 2015, and 2016 (Table 1). Repartition of symptomatic plants among the above-described disease severity classes (Fig. 1a–d) was very variable year by year, with a prevalence of mild symptoms in 2015 and 2016 seasons.

**Table 1:** GPG disease prevalence, incidence, and symptom severity in the vineyard studied during the periods 2013–2016

		2013 (%)	2014 (%)	2015 (%)	2016 (%)
GPG disease presence		90	100	100	100
GPG disease prevalence*		64.0	30.4	14.1	13.2
GPG disease incidence**		/	9.2	0	0.9
Symptom repartition during the observation period	Severe	12.9	75.0	31.2	20.0
	Moderate	27.3	8.3	18.7	6.7
	Mild	59.8	16.7	50.1	73.3

*Prevalence: percentage of symptomatic plants in a given year

**Incidence: percentage of newly symptomatic plants in a given year; / = data not available

In addition to general leaf symptom phenotypes ascribable to viral diseases, such as chlorotic leaf mottling, leaf deformation, and puckering, the symptomatic grapevines also showed the complete set of symptoms specifically associated with GPG-disease that are widely described in the literature (Fig. 1e and Giampetruzzi et al. 2012).

### Detection of GPGV and other grapevine viruses

A total of 25 grapevine samples, 20 from field and five from greenhouse, were tested for the presence of GPGV using real-time RT-PCR with specific primers GPgV504-F and GPgV588-R.

GPGV was found in all field-grown grapevines independently of symptom presence and severity. The Ct values ranged from 29.77 to 34.85 (Table 2). Samples from greenhouse-grown grapevines tested negative to GPGV, since their Ct values were greater than 35 or not classified (Bianchi et al. 2015; Vončina et al. 2017).

**Table 2:** Detection of GPGV in Pinot gris samples by real-time RT-PCR approach using GPGV-504 F- / GPGV-588 R-specific primers

Plant condition	Symptoms	Sample ID	GPGV				
			Ct value	result	Average Ct value	SD	Statistical analyses°
*Pinot gris* greenhouse-grown grapevine controls	–	1-ctrl	Nd	–	–	–	–
	–	2-ctrl	Nd	–			
	–	3-ctrl	Nd	–			
	–	4-ctrl	Nd	–			
	–	5-ctrl	Nd	–			
*Pinot gris* field-grown grapevines	Asymptomatic	1-as	32.74	+	33.38	0.85	*** in comparison with sv–plants
	Asymptomatic	2-as	34.50	+			
	Asymptomatic	3-as	33.38	+			
	Asymptomatic	4-as	32.41	+			
	Asymptomatic	5-as	33.89	+			
	Mild	1-ml	34.85	+	33.74	0.71	*** in comparison with sv–plants
	Mild	2-ml	33.87	+			
	Mild	3-ml	32.91	+			
	Mild	4-ml	33.64	+			
	Mild	5-ml	33.45	+			
	Moderate	1-md	31.51	+	32.60	0.64	*** in comparison with sv–plants
	Moderate	2-md	32.78	+			
	Moderate	3-md	33.21	+			
	Moderate	4-md	32.84	+			
	Moderate	5-md	32.65	+			
	Severe	1-sv	30.93	+	30.72	0.88	*** in comparison with as– and ml– plants ** in comparison with md–plants
	Severe	2-sv	31.05	+			
	Severe	3-sv	29.94	+			
	Severe	4-sv	31.91	+			
	Severe	5-sv	29.77	+			

as–plants = asymptomatic plants; ml–plants = plants showing mild symptoms; md–plants = plants showing moderate symptoms; sv–plants = plants showing severe symptoms; nd = virus not detected

**°**Family-wise significance and confidence level: 0.05

*P < 0.05

**P < 0.01

***P < 0.001

Real-time RT-PCR and ELISA analyses excluded the presence of GVA, GVB, GFLV, ArMV, GFkV, GLRaV-1, GLRaV-2, GLRaV-3, thus viruses included in the Italian certification program. Conversely, our real-time RT-PCR assay detected the ubiquitous presence of GRSPaV, HSVd, and GYSVd-1, both in field- and greenhouse-grown grapevines, showing Ct values significantly lower than 30 (Supplementary Table 1).

### Transmission electron microscopy

TEM observations were performed on leaf tissues collected from plants from field and from greenhouse, previously tested by nucleic acid-based and serological methods.

#### Ultrastructure of tissues from greenhouse-grown control grapevines

**Fig. 2:**
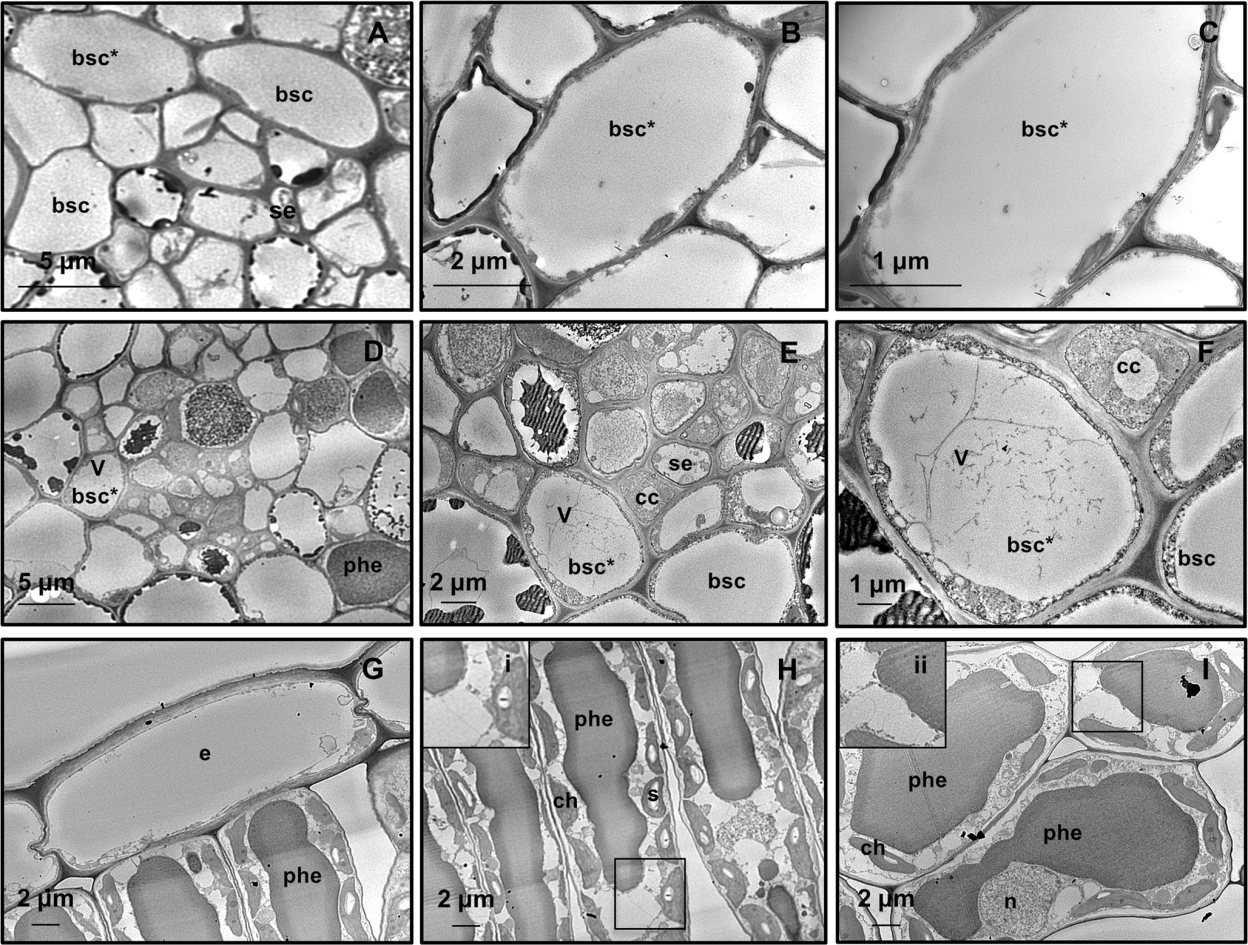
Representative TEM micrographs of grapevine leaf tissues. **a** In greenhouse-grown control leaf tissue, the bundle sheath cells appear preserved. **b, c** Virus particles are not present, as evidenced by the observation at higher magnifications. **d**–**i** Independent of the presence or severity of symptoms, tissues from all field-grown grapevines host virus-like particles. **d**–**f** In the vein, a bundle sheath cell contains numerous filamentous, flexuous virus-like particles in the vacuole. **g**–**i P**articles are not present in the epidermis (**g**), in the palisade (**h**), and in the spongy parenchyma (**i**). In insets (i) and (ii) vacuolar areas are magnified. In **a, b,** and **c** asterisks * indicate the same cell at progressive magnification. (bsc, bundle sheath cell; cc, companion cells; ch, chloroplast; e, epidermis; n, nucleus; phe, vacuolar phenolics; se, sieve element; s, starch; v, virus-like particles)

Virus particles were not detected in greenhouse-grown control grapevines. Phloem tissue and, in particular, bundle sheath cells (BSCs), which are located as a ring-like sleeve around the vascular bundle, showed their typical organization (for review see Staehelin 2015) (Fig. 2a–c).

#### Virus morphology and localization in field-grown grapevine leaf tissues

**Fig. 3:**
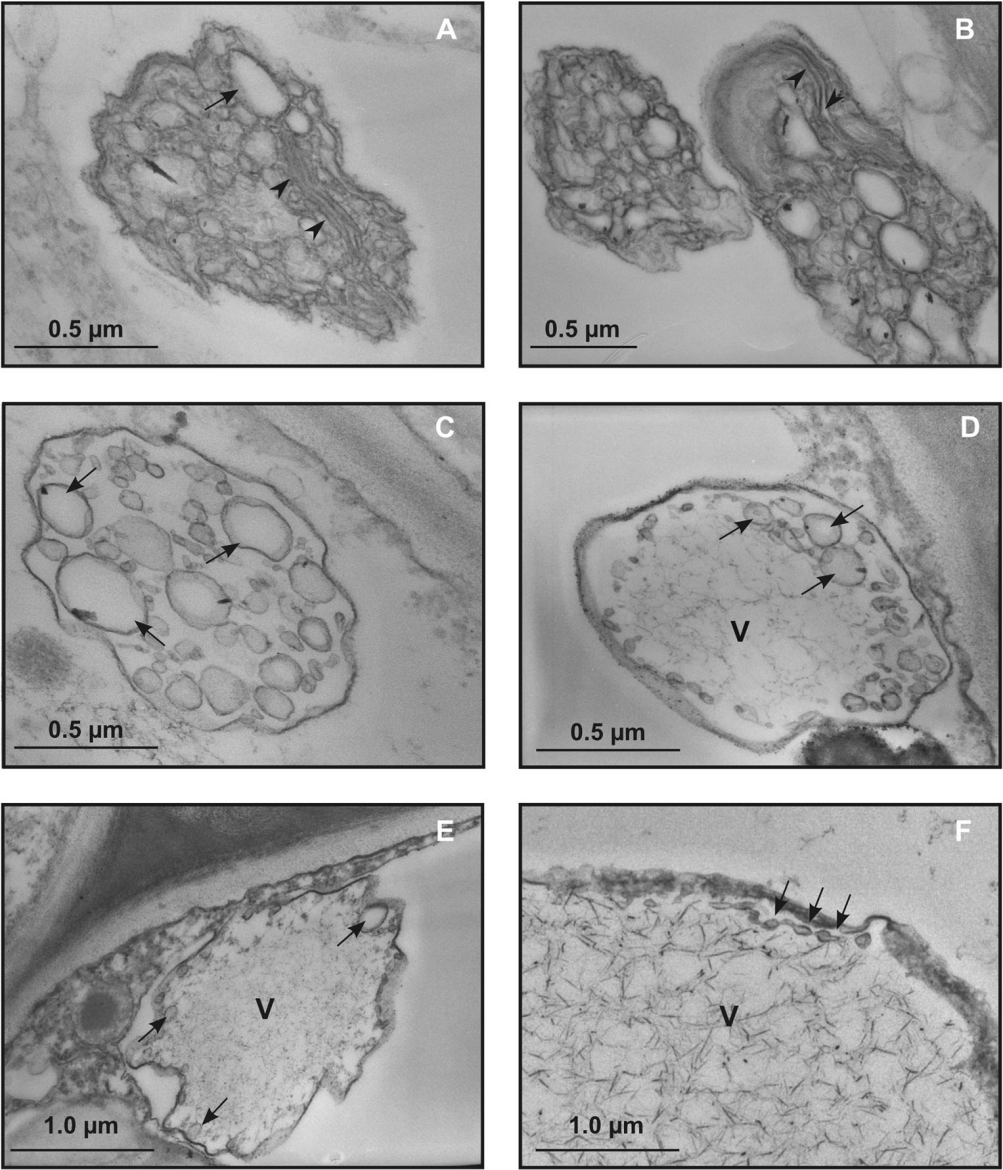
Representative TEM micrographs of leaf tissue from field-grown grapevines. **a**–**f** Independent of the presence or severity of symptoms, in the bundle sheath cells, membrane-bound organelles are present. **a**, **b** Membrane-bound organelles contain vesicles (arrow) and flattened membrane disks (arrow-heads). **c**, **d**, **e**, **f** Membrane-bound organelles contain large globular vesicles alone (**c,** arrows) or vesicles (**d**, **e**, **f**, arrows) and filamentous virus-like particles. In the latter cases, vesicles are localized at the organelle periphery. (v, virus-like particles)

Filamentous flexuous virus-like particles, not arranged in bundles, were detected in samples from all plants grown in the field, independent of symptom presence and severity (Figs. 2d–f; 3d–f). The particles were observed in the BSCs (Fig. 2d–f), but not in the epidermis (Fig. 2g) nor in palisade (Fig. 2h) and spongy parenchyma (Fig. 2i). Viruses were in the vacuoles (Fig. 2d–f) or inside membrane-bound structures (Fig. 3d–f).

#### Ultrastructural modifications in field-grown grapevine leaf tissues

The following ultrastructural modifications occurred in leaf tissues from all field-grown grapevines tested in this work, independent of symptom presence and severity.

**Fig. 4:**
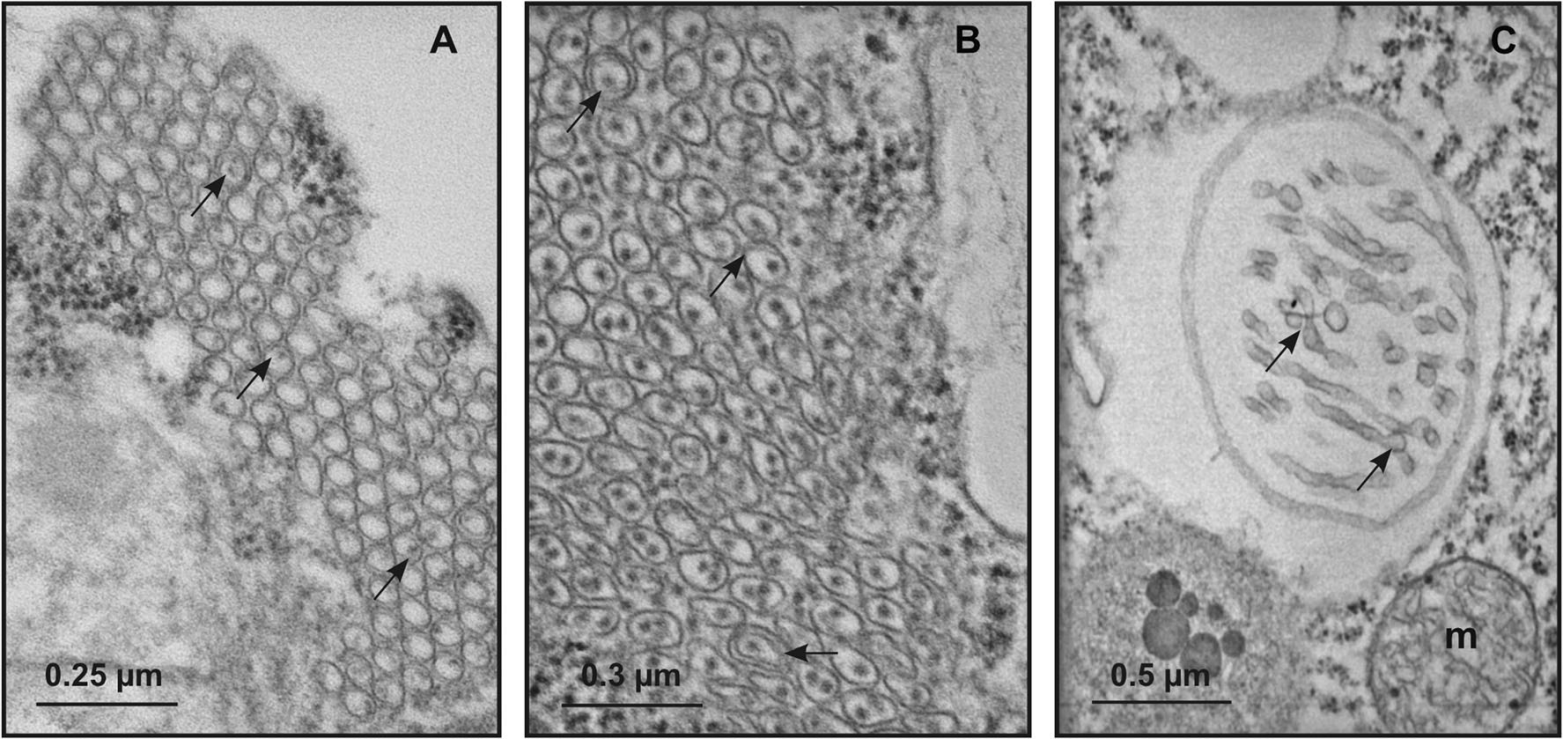
Representative TEM micrographs of leaf tissue from field-grown grapevines. **a**, **b** Single- and double-membraned rounded vesicles containing finely granular structures (arrows) and organized into packets are also observed in bundle sheath cells from leaf samples of all field-grown grapevines. **c** Alongside normal-shaped mitochondrion, enlarged mitochondrion with swollen cristae (arrows) is found in mesophyll cells. (m, mithochondrion)

**Fig. 5:**
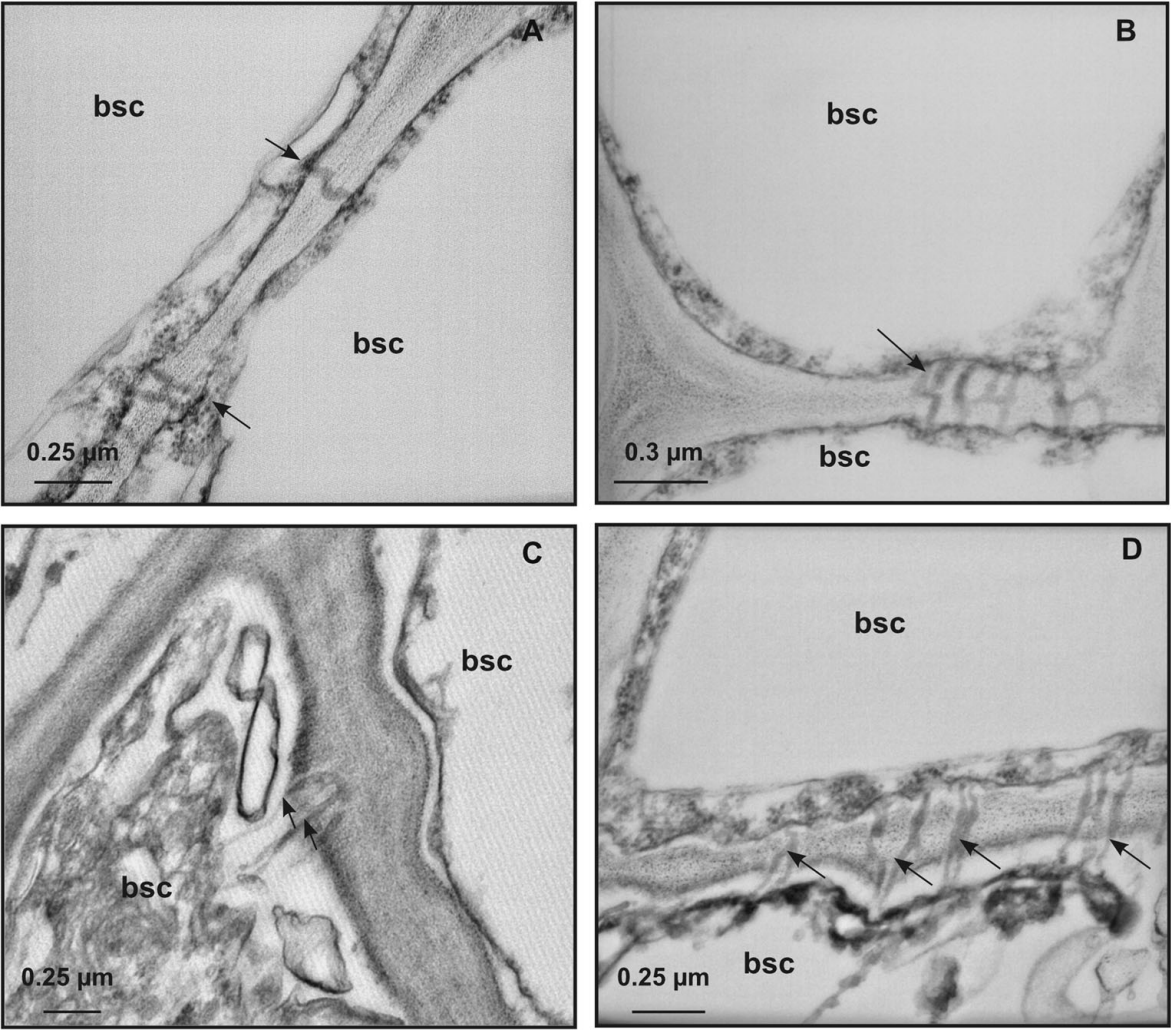
Representative TEM micrographs of leaf tissue from greenhouse- (**a**) and field-grown grapevines (**b**–**d**). **a** Plasmodesmata (arrows) display normal shape and size. **b** plasmodesmata (arrow), normal in shape and size, connect non-ultrastructurally altered bundle sheath cells. **c**, **d** Plasmodesmata connecting ultrastructurally altered to adjacent non-ultrastructurally altered bundle sheath cells display extended tubular terminal arrangements protruding into the cell lumen (arrows). (bsc, bundle sheath cells)

In BSCs, membrane-bound organelles were observed (Fig. 3). They showed distorted flattened membrane disks (Fig. 3a, b) and/or contained numerous vesicles grouped in packets (Fig. 3c), often located in the peripheral zone (Fig. 3d–f). The vesicles were globular in shape and displayed polymorphism, with diameters ranging between 13.0 and 30.0 nm (Fig. 3c, d). Inside such structures, there were accumulations of virus-like filamentous particles (Fig. 3d–f). Patches of single- and double-membraned-rounded vesicles, containing finely granular material (Fig. 4a, b), were also observed in BSCs from leaf samples of all field-grown grapevines. The nature of these vesicular arrangements was not determined. None of the above-described structures were observed in leaves from control grapevines grown in the greenhouse (Fig. 2a–c). As in healthy plants (Fig. 5a), plasmodesmata connecting ultrastructurally non-altered BSCs (Fig. 5B) showed simple or H-shaped longitudinal profiles (for review see Roberts and Oparka 2003). On the other hand, plasmodesmata connecting those BSCs to the adjacent ultrastructurally modified BSCs presented extended terminal structures protruding into the cell lumen (Fig. 5c, d).

All the above-described ultrastructural modifications were neither present in epidermis (Fig. 2g) nor in mesophyll (Fig. 2h, i). In mesophyll cells, some mitochondria appeared modified in the infected samples, showing enlargement and vesiculation (Fig. 4c), while chloroplasts and nuclei displayed a normal morphology (Fig. 2h, i).

### Immuno-cytochemical identification of GPGV in grapevine tissues

**Fig. 6:**
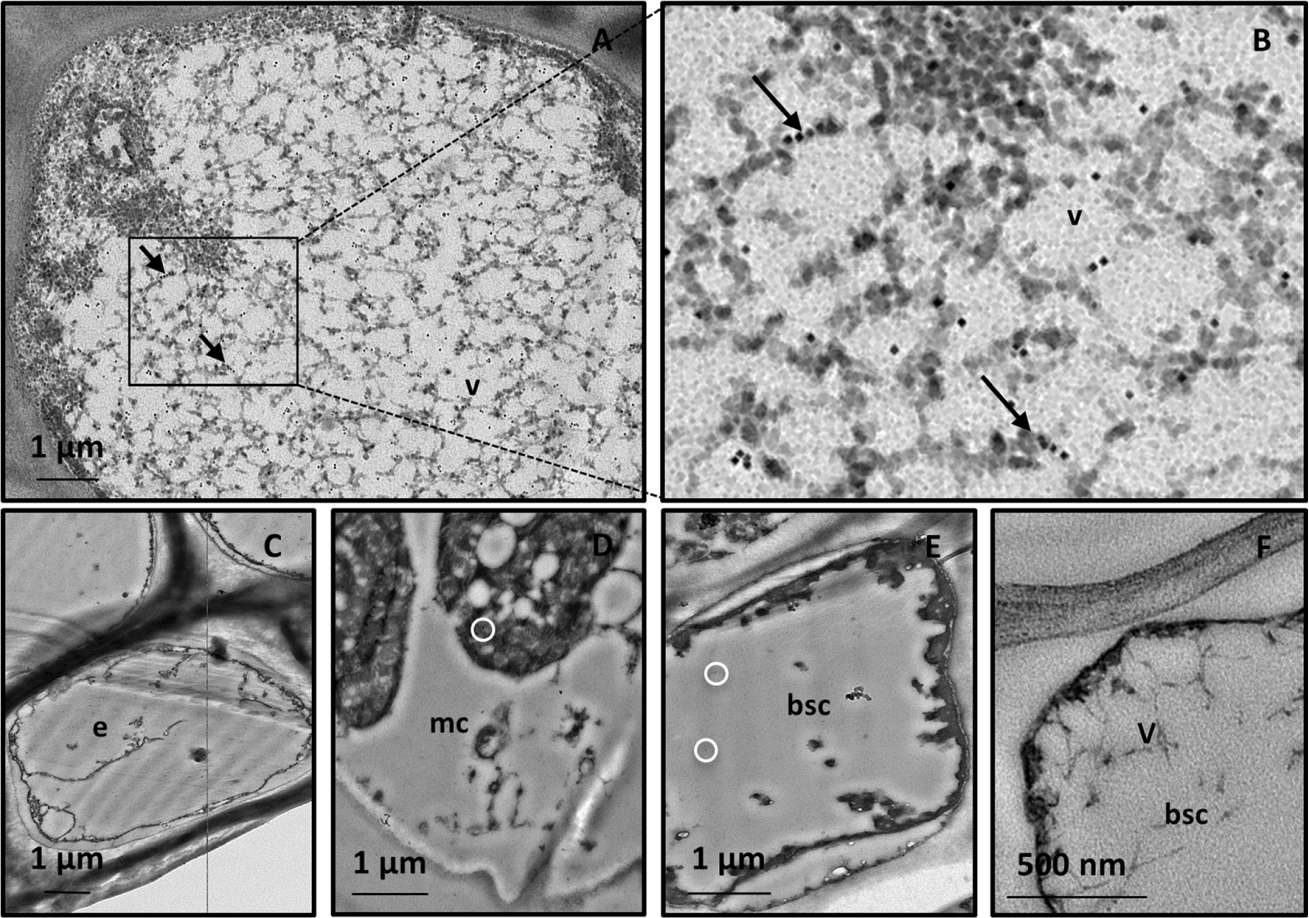
Representative TEM micrographs of immunogold-labeled grapevine tissues. **a, b** In samples incubated with dilution 1:10 of primary rabbit polyclonal antibody (Pab) against GPGV-coat protein and dilution 1:50 of secondary gold-conjugated antibody, gold (arrows) is visible in the bundle sheath cells of field-grown grapevines, in association with the filamentous particles and in their proximity. **c**–**e** In epidermis (**c**) and mesophyll cells (**d**) from field-grown grapevines, as well as in bundle sheath cells from greenhouse-grown grapevines (**e**), gold labelling was negligible or absent after incubation with Pab against GPGV-coat protein (circles in **d** and **e**). **f** Label does not occur in infected samples incubated with buffer alone. (bsc, bundle sheath cell; e, epidermis; mc, mesophyll cell; v, virus-like particles)

Immuno-cytochemical analyses revealed positive reaction of the anti-GPGV-CP Pab with the virus-like filamentous structures observed in BSCs (Fig. 6). Using the dilutions 1:10 of Pab and 1:50 of GAR, gold was detected on the filamentous particles (Fig. 6a, b). No label occurred in epidermis (Fig. 6c) and mesophyll cells (Fig. 6d). Samples from greenhouse-grown grapevines (Fig. 6e) and infected samples incubated with buffer alone (Fig. 6f) did not show labelling.

## Discussion

The absence of the viruses included in the Italian certification program (GVA, GVB, GFLV, ArMV, GFkV, GLRaV-1, GLRaV-2, and GLRaV-3, Bertazzon et al. 2002), and the ubiquitous presence of GRSPaV and grapevine viroids HSVd and GYSVd-1 (Martelli et al. 2007; Meng et al. 2006), both in the field- and greenhouse-grown control grapevines, allowed us to focus on GPGV-plant interactions.

The results of real-time RT-PCR and ELISA analyses carried out in this work support the preliminary observations (Saldarelli et al. 2015) that lack of visible disease symptoms (Giampetruzzi et al. 2012) does not necessarily indicate the absence of GPGV in field-grown Pinot gris. Interestingly, virus association with symptomless host plants is a trait previously described for GINV (Nishijima et al. 2000) and for some other filamentous plant viruses of the family *Betaflexiviridae* and *Closteroviridae* (Gattoni et al. 2009).

The variety of symptoms observed in the vineyard and the association between symptom severity and virus titers suggest diversity of GPVG virulence and spread efficiency (Saldarelli et al. 2015; Bertazzon et al. 2016b; Tarquini et al. 2016). Furthermore, plant and environmental factors could also play a role in symptom development in Pinot gris in the field, as reported for other plant/virus interactions (Cecchini et al. 1998).

Cytological analyses showed the exclusive presence of filamentous flexuous virus-like particles in leaf tissues of the 20 field-grown grapevines. Particles were present in the deep parenchyma, a trait similar to that reported for GINV, visualized in the phloem parenchyma (Yoshikawa et al. 1997), and other *Flexiviridae* (Saldarelli et al. 2008).

The filamentous flexuous particles were observed and identified by immunogold labelling in the field-grown grapevines, but not in those grown in the greenhouse, supporting the evidence that they are GPGVs and not other filamentous viruses, such as GRSPaV, ubiquitous distributed in grapevines (Martelli et al. 2007; Meng et al. 2006).

The localization of the virus particles in the deep parenchyma cells could be compatible with the possibility that the vector is the grapevine eriophyoid mite *Colomerus vitis*, as suggested by Malagnini et al. (2016). *C. vitis* overwinters on grapevine as adult, feeding, in spring, on the young leaf buds (Duso et al. 2012). Thus, it is likely that the mite is able to pierce the deeper tissues, in spite of its short stylet (Chetverikov 2015). The fact that the virus is localized in the deep part of the leaf tissues also matches with the failures in sap transmissions (Malagnini et al. 2016). Interestingly, GINV, which is closely related to GPGV (Yoshikawa et al. 1997), is transmitted by *C. vitis* (Kunugi et al. 2000).

Immunogold labelling, performed using a specific polyclonal antibody (Pab) previously tested in western blot analyses against the GPGV-CP (Gualandri et al. 2015), supports our conclusion that the filamentous virus-like particles observed in grapevine BSCs are GPGV. The presence of gold in proximity of the particles could be due to the diffuse distribution of the CP in the infected cells and/or to the fact that, in ultrathin sections, elongated viruses are often detected as fragments of the whole particles, reducing the labelling accuracy (Milne 1992). The specificity of the Pab/virus reaction is here further supported by the absence of signal in cells different from that of BSCs, as well as in greenhouse-grown control grapevines.

Even in absence of visible disease symptoms, ultrastructural modifications were present in all the field-grown grapevines (Cecchini et al. 1997), but not in greenhouse-grown plants. The most evident modifications were in the BSCs, which displayed membrane-bound structures containing flattened disks and/or vesicles, very similar to those observed in other virus/plant host interactions, as deformed endoplasmic reticulum (ER, Bamunusinghe et al. 2011).

The presence of membrane-bound organelles, often containing virus particles, allowed us to hypothesize a possible role in GPGV replication or assembly, even if in this study, the nature of these organelles was not determined. It is well demonstrated that animal and plant viruses cause in the host cell the formation of membranous structures, derived from the alterations of different cell organelles, with a high degree of specificity to the virus taxonomic group (Laliberté and Sanfaçon 2010). These new-formed membranous structures are known as “virus factories” (Miller and Krijnse-Locker 2008), which, interacting with viral and host proteins originate the so-called viral replication complexes (VRCs, Hyodo et al. 2014) The formation of the VRCs involves multiplex interactions and signals between viral and cell factors. Mitochondria, cell membranes, and cytoskeleton frequently participate in the biogenesis of VRCs, supplying energy and other essential factors for the viral replication cycle (Fernandez de Castro et al. 2013).

Most positive single-strand RNA viruses form VRCs in association with ER, but which cell membranes are utilized by *Betaflexiviridae* for replication, have not been clarified yet. Recently, the association of the replicase protein with the host-cell ER was reported for GRSP-aV (Prosser et al. 2015).

Plasmodesmata connecting BSCs to neighboring yet non-modified cells showed ultrastructural modifications in samples from field-grown grapevines. The cell-to-cell and systemic transport is associated with plasmodesma functional and, in some cases, morphological modifications (Choi 1999; Stewart et al. 2009). So far, two basic principles for cell-to-cell movement of plant viruses have been described: tubule-guided movement of intact virions or non-tubule-guided movement as ribonucleoprotein complexes (Lazarowitz and Beachy 1999). Isometric viruses such as *Cauliflower Mosaic Virus*, *Tomato Spotted Wilt Virus*, *Cowpea Mosaic Virus*, and viruses belonging to *Nepoviridae* adopted the first mode and move through plasmodesmata using tubular structures induced by the viral movement proteins (MPs) (Benitez-Alfonso et al. 2010). The spread of *Tobacco mosaic virus* and several other viruses (Sambade and Heinlein 2009; Epel 2009) occurs by the interactions among viral RNA, MPs, actin cytoskeleton, cell microtubules, and ER surface (Niehl et al. 2013).

Plasmodesmata connecting two ultrastructurally altered BSCs in GPGV-infected samples presented extended terminal protrusions very different from the tubules described for the above-cited isometric viruses (McMullen et al. 1977). Even if the nature of such protrusions is not determined in this work, a role of these protrusions in GPGV cell-to-cell movement might be hypothesized.

In conclusion, this work confirmed that GPGV is present in grapevines showing different symptom phenotypes in the vineyard: this calls for further investigations to clarify GPG-disease etiology. Our results showed that GPGV is located in grapevine deep parenchyma cells, the BSCs. We provided evidence that virus-specific ultrastructural modifications are elicited by the infection and that they can be of diagnostic value, even in asymptomatic plants.

Due to the increase in reports of GPG-disease worldwide and the scarcity of knowledge about the *Betaflexiviridae* family, a description of GPGV-associated ultrastructural modifications will have important implications, both scientifically and economically, regarding disease management and control. Given the difficulties of working with field-grown woody plants, the establishment of model experimental systems will be necessary for the functional study of the ultrastructural modifications described in this work.

## Electronic supplementary material

Supplementary table 1 (DOCX 131 kb)
