# Supplementary material for: Localization and subcellular association of Grapevine Pinot Gris Virus in grapevine leaf tissues
Source: Protoplasma. 2017 Dec 22;255(3):923–35. doi: 10.1007/s00709-017-1198-5 (PMC5904240; doi:10.1007/s00709-017-1198-5)
Supplement: Supplementary file 1 — (DOCX 131 kb) [file 709_2017_1198_MOESM1_ESM.docx]

**Supplementary table 1.** Serological and molecular detection of other viruses in Pinot gris samples object of the present study.

| Plant condition | Symptoms | Sample ID | ELISA | | | | | | | | One-step Multiplex RT-qPCR | | | | | | | | | |
| --- | --- | --- | --- | --- | --- | --- | --- | --- | --- | --- | --- | --- | --- | --- | --- | --- | --- | --- | --- | --- |
|  |  |  | GVA | GVB | GFLV | ArMV | GFkV | GLRaV-1 | GLRaV-2 | GLRaV-3 | GVA | GFLV | ArMV | GLRaV-1 | GLRaV-3 | GRSPaV | GYSVd-1 | HSVd | GRVFV | GSyV-1 |
| *Pinot gris*  *Greenhouse-grown control plants* | - | 1-ctrl | - | - | - | - | - | - | - | - | nd | nd | nd | nd | nd | 22.62 | 25.63 | 24.28 | nd | nd |
|  | - | 2-ctrl | - | - | - | - | - | - | - | - | nd | nd | nd | nd | nd | 22.69 | 24.00 | 22.40 | nd | nd |
|  | - | 3-ctrl | - | - | - | - | - | - | - | - | nd | nd | nd | nd | nd | 21.22 | 24.38 | 21.79 | nd | nd |
|  | - | 4-ctrl | - | - | - | - | - | - | - | - | nd | nd | nd | nd | nd | 22.66 | 24.30 | 22.99 | nd | nd |
|  | - | 5-ctrl | - | - | - | - | - | - | - | - | nd | nd | nd | nd | nd | 22.08 | 25.19 | 24.55 | nd | nd |
| *Pinot gris*  *Field-grown plants* | asymptomatic | 1-as | - | - | - | - | - | - | - | - | nd | nd | nd | nd | nd | 22.19 | 24.07 | 24.32 | nd | nd |
|  | asymptomatic | 2-as | - | - | - | - | - | - | - | - | nd | nd | nd | nd | nd | 21.36 | 24.67 | 22.52 | nd | nd |
|  | asymptomatic | 3-as | - | - | - | - | - | - | - | - | nd | nd | nd | nd | nd | 23.53 | 24.22 | 21.85 | nd | nd |
|  | asymptomatic | 4-as | - | - | - | - | - | - | - | - | nd | nd | nd | nd | nd | 24.59 | 25.19 | 23.11 | nd | nd |
|  | asymptomatic | 5-as | - | - | - | - | - | - | - | - | nd | nd | nd | nd | nd | 24.18 | 26.25 | 24.53 | nd | nd |
|  | mild | 1-ml | - | - | - | - | - | - | - | - | nd | nd | nd | nd | nd | 23.48 | 24.05 | 23.78 | nd | nd |
|  | mild | 2-ml | - | - | - | - | - | - | - | - | nd | nd | nd | nd | nd | 22.38 | 24.55 | 23.90 | nd | nd |
|  | mild | 3-ml | - | - | - | - | - | - | - | - | nd | nd | nd | nd | nd | 21.61 | 24.22 | 24.19 | nd | nd |
|  | mild | 4-ml | - | - | - | - | - | - | - | - | nd | nd | nd | nd | nd | 23.77 | 25.82 | 26.20 | nd | nd |
|  | mild | 5-ml | - | - | - | - | - | - | - | - | nd | nd | nd | nd | nd | 24.93 | 26.11 | 23.21 | nd | nd |
|  | moderate | 1-md | - | - | - | - | - | - | - | - | nd | nd | nd | nd | nd | 22.05 | 25.06 | 23.07 | nd | nd |
|  | moderate | 2-md | - | - | - | - | - | - | - | - | nd | nd | nd | nd | nd | 20.44 | 24.23 | 22.12 | nd | nd |
|  | moderate | 3-md | - | - | - | - | - | - | - | - | nd | nd | nd | nd | nd | 20.25 | 24.75 | 23.15 | nd | nd |
|  | moderate | 4-md | - | - | - | - | - | - | - | - | nd | nd | nd | nd | nd | 23.28 | 24.33 | 22.75 | nd | nd |
|  | moderate | 5-md | - | - | - | - | - | - | - | - | nd | nd | nd | nd | nd | 21.24 | 26.05 | 22.41 | nd | nd |
|  | severe | 1-sv | - | - | - | - | - | - | - | - | nd | nd | nd | nd | nd | 21.3 | 25.87 | 23.10 | nd | nd |
|  | severe | 2-sv | - | - | - | - | - | - | - | - | nd | nd | nd | nd | nd | 21.18 | 20.41 | 21.80 | nd | nd |
|  | severe | 3-sv | - | - | - | - | - | - | - | - | nd | nd | nd | nd | nd | 22.11 | 24.48 | 23.36 | nd | nd |
|  | severe | 4-sv | - | - | - | - | - | - | - | - | nd | nd | nd | nd | nd | 22.25 | 24.43 | 22.87 | nd | nd |
|  | severe | 5-sv | - | - | - | - | - | - | - | - | nd | nd | nd | nd | nd | 23.58 | 23.72 | 22.51 | nd | nd |

GVA (*Grapevine Virus A*); GVB (*Grapevine Virus B*); GFLV (*Grapevine Fanleaf Virus*); ArMV (*Arabis Mosaic Virus*); GFkV (*Grapevine Fleck Virus*); GLRaV-1,2,3 (*Grapevine Leafroll-associated Viruses* 1,2,3); GRSPaV (*Grapevine Rupestris Stem Pitting-associated Viruses*); GYSVd-1 (*Grapevine Yellow Speckle Viroid* 1); HSVd (*Hop Stunt Viroid*); GRVFV (*Grapevine Rupestris Vein Feathering Virus*) and GSyV-1 (*Grapevine Syrah Virus* 1).

as-plants = asymptomatic plants; ml–plants = plants showing mild symptoms; md–plants = plants showing moderate symptoms; sv–plants = plants showing severe symptoms.

nd = virus not detected
